# Supplementary material for: Synchronized mesenchymal cell polarization and differentiation shape the formation of the murine trachea and esophagus
Source: Nat Commun. 2018 Jul 19;9:2816. doi: 10.1038/s41467-018-05189-2 (PMC6053463; doi:10.1038/s41467-018-05189-2)
Supplement: Supplementary file 1 — Supplementary Information [file 41467_2018_5189_MOESM1_ESM.pdf]

Supplementary Information for

Synchronized mesenchymal cell polarization and differentiation shape the formation of murine trachea and esophagus

by Kishimoto et al.,

Supplementary Table 1 Primary antibody list and conditions for immunofluorescence.

Antibody, Dilution	Company, Catalog code	Fixative	Tissue Preparation	Antigen retrieval	Secondary antibody, Amplification
phospho-Histon H3 (1:100)	Santa Cruz Biotechnology, Inc, sc-12927	4% PFA	Paraffin	105°C for 15 min in Histo^{VT} One	Rabbit-Alexa488
CDH1 (1:800)	Cell Signaling Technology, #3195	4% PFA	Paraffin	105°C for 15 min in Histo^{VT} One	Rabbit-Alexa488
β-catenin (1:200)	Cell Signaling Technology, #8847	4% PFA	Paraffin	105°C for 15 min in Histo^{VT} One	Mouse-Alexa488
Phospho-MLC2 (Ser 19)	Cell Signaling Technologies, ell Signaling Technologies, #3675	4% PFA	Paraffin	105°C for 15 min in Histo^{VT} One	Mouse-Alexa488
Phospho-MLC2 (Thr19/Ser19) (1/200)	Cell Signaling Technology, #3674	4% PFA	Paraffin	105°C for 15 min in Histo^{VT} One	Rabbit-Alexa488
Cy3-SMA (1:1,000)	Sigma-Aldrich, C6198	4% PFA	Paraffin	105°C for 15 min in Histo^{VT} One	
SM22α (1:1,000)	Abcam, ab14106	4% PFA	Paraffin	105°C for 15 min in Histo^{VT} One	Rabbit-Alexa594
GM130 (1:100)	BD Bioscences, 610822	4% PFA	Paraffin	105°C for 15 min in Histo^{VT} One	Mouse-Alexa488
γ-tubulin (1/100)	Abcam, Ab11321	4% PFA	Paraffin	105°C for 15 min in Histo^{VT} One	Rabbit-Alexa647
Sox9 (1/100)	MILLIPORE, AB5535	4% PFA	Paraffin	105°C for 15 min in Histo^{VT} One	Rabbit-Alexa488
Foxj1 (1/100)	Gift from Dr. Steven L. Brody	4% PFA	Paraffin	105°C for 15 min in Histo^{VT} One	Mouse-Alexa488
CC10 (T-18) (1/400)	Santa Cruz Biotechnology, Inc, sc-9772	4% PFA	Paraffin	105°C for 15 min in Histo^{VT} One	Goat-Alexa594
Krt5 (1/800)	Abcam, Ab24647	4% PFA	Paraffin	105°C for 15 min in Histo^{VT} One	Rabbit-Alexa647
GFP (1/200)	Thermo Fisher Scientific, A10262	4% PFA	Frozen	90°C for 5 min in Histo^{VT} One	Chicken-Alexa488

Supplementary Figure 1

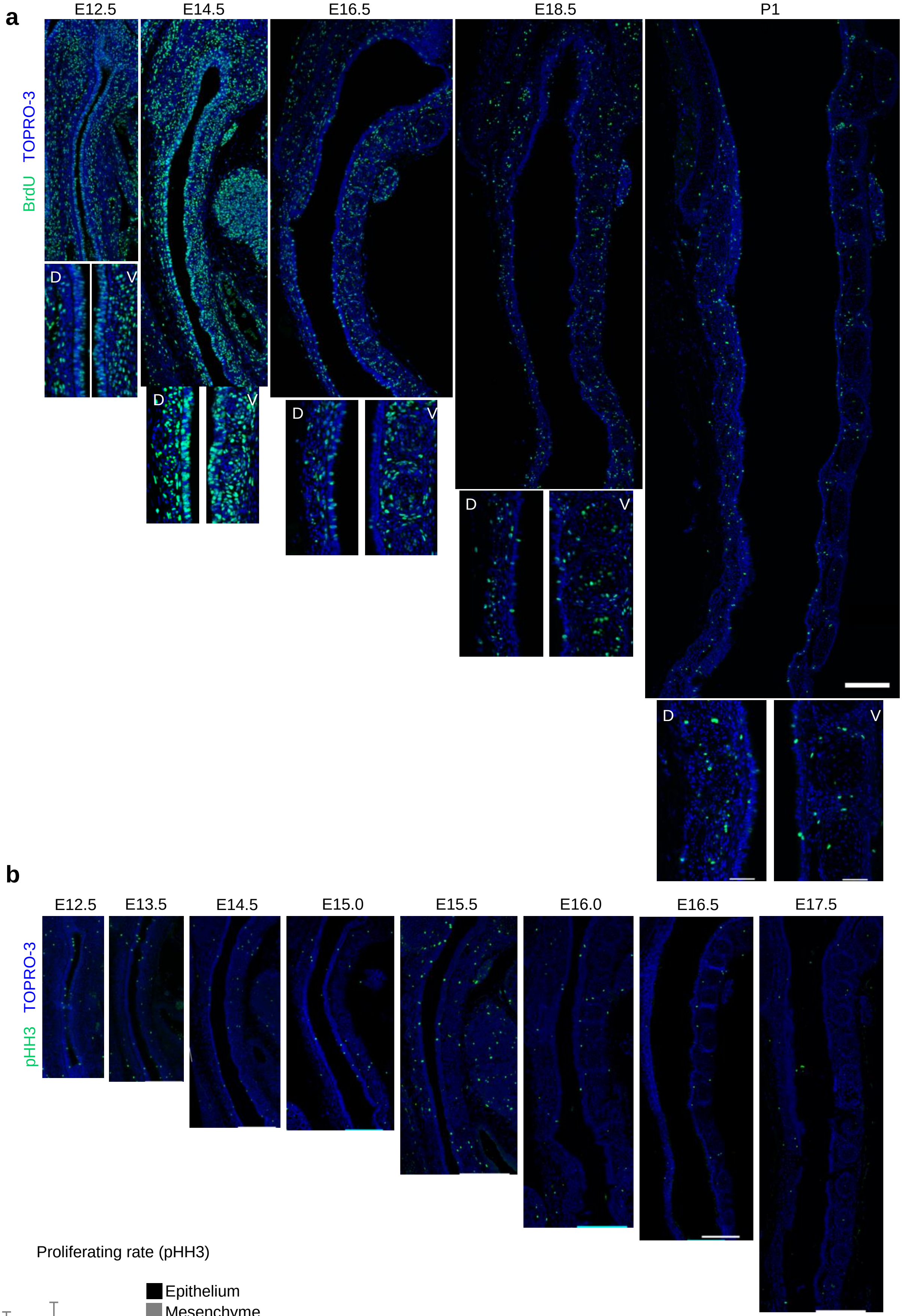

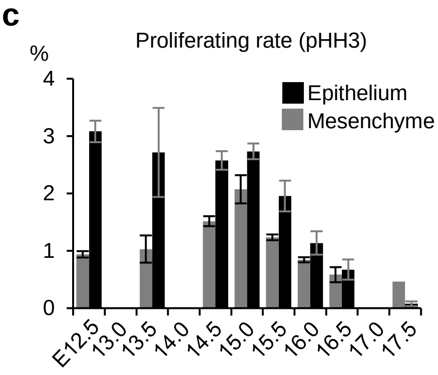

Supplementary Figure 1 Cell proliferation during embryogenesis. (**a**) Distribution of BrdU-incorporated cells in developing trachea. Sections were stained for BrdU (*Green*) and TOPRO-3 (*Blue*). Magnified views are shown below. (**b**) Distribution of pHH3⁺ cells in developing trachea. Sections were stained for pHH3 (*Green*) and TOPRO-3 (*Blue*). (**c**) Proportion of proliferating cells determined by pHH3 expression. D, Dorsal; V, Ventral. Data represent means ± SEM (n≧4). Scale bar; 200 μm (**a, b**), 50 μm (**a;** *Magnified view*).

Supplementary Figure 2

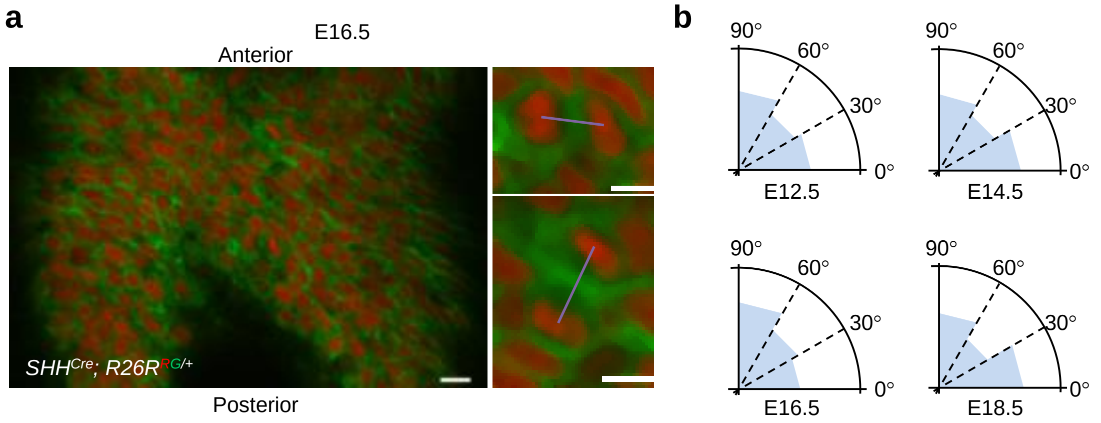

Supplementary Figure 2 Orientation of cell division in epithelium during embryogenesis. (**a**) Representative images of cell division in *SHH*^{Cre}*; R26R*^{RG} tracheal epithelium at E16.5. Red indicates nuclei genetically labeled by H2B-mCherry. Green indicates plasma membrane labeled by EGFP-GPI. (**b**) Quantification of cell division orientation based on (**a**). 0˚ corresponds to clockwise circumferential direction. 90˚ corresponds to anterior direction. (n = 30, 37, 35, and 23 in more than three embryos each for E12.5, 14.5, 16.5 and 18.5). Scale bar; 10 μm.

Supplementary Figure 3

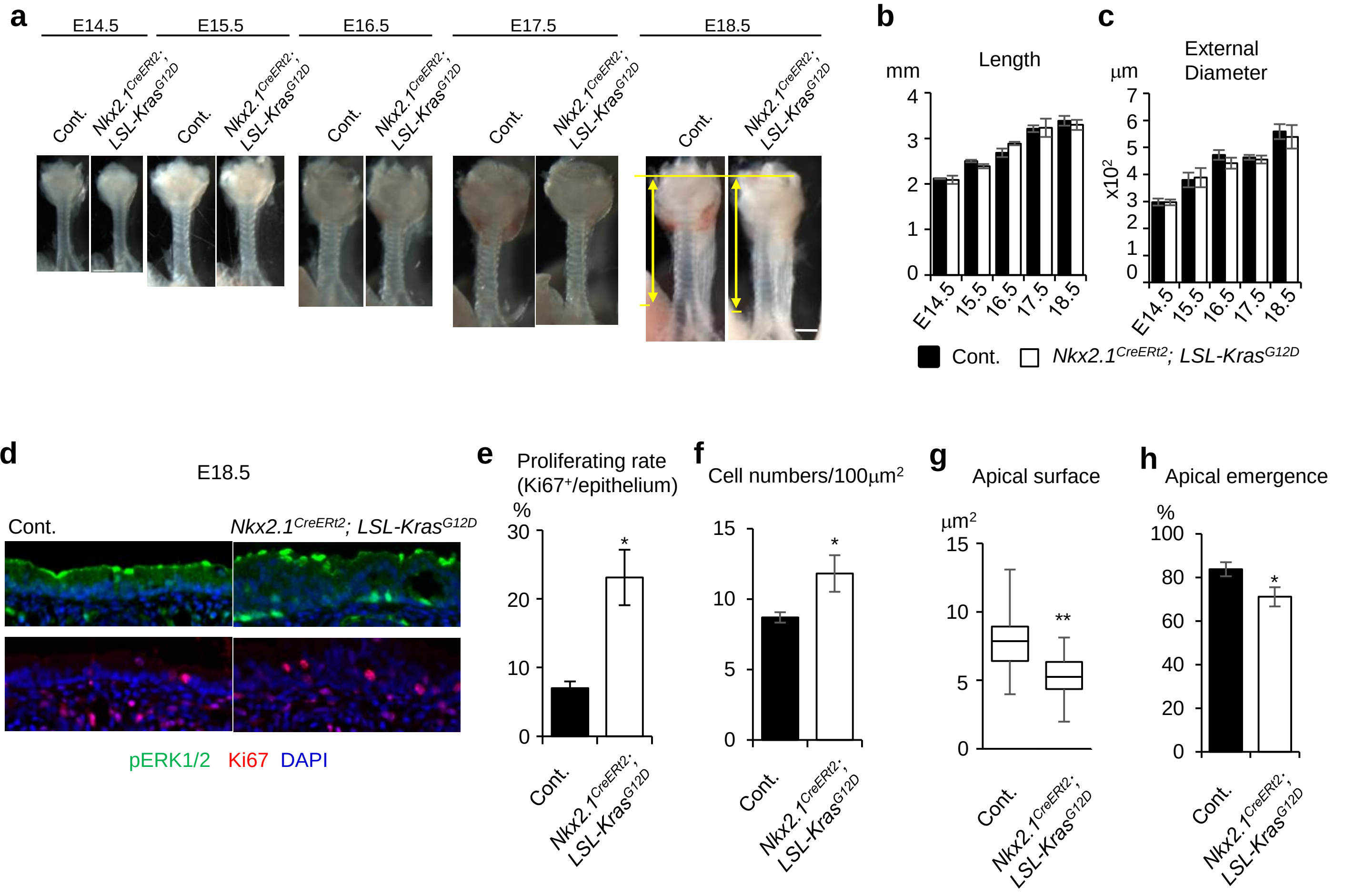

Supplementary Figure 3 Ectopic epithelial cell proliferation in phase 2 inhibited epithelial rearrangement, but neither tube elongation nor expansion.

(**a**) Gross morphology of developing *Nkx2.1*^{CreERt2}*, LSL-Kras*^{G12D} trachea. Tube length (**b**). External diameter (**c**). Data represent means ± SEM (n≧3). (**d**) Ki67 expression and phosphorylated ERK1/2 in *Nkx2.1*^{CreERt2}*, LSL-Kras*^{G12D} trachea. Sections were stained for pERK1/2 (*Green*), Ki67 (*Red*), and DAPI (*Blue*). (**e**) Rate of proliferating epithelial cells determined by Ki67 expression at E18.5. Data represent means ± SEM (n=3). (**f**) The number of GFP^{+} epithelial cells per 100µm^{2}. Data represent means ± SD (Control, n = 123, *Nkx2.1*^{CreERt2}*; LSL-Kras*^{G12D}, n =167 in 3 fields, respectively) . (**g**) Apical surface area of GFP^{+} epithelial cells at E18.5. Centre line, box limits and whiskers represent mean, 25 and 75% confidence limits, and min and max values, respectively. (Control, n = 52, *Nkx2.1*^{CreERt2}*; LSL-Kras*^{G12D}, n =50) (**h**) Proportion of luminal cells in GFP^{+} epithelial cells. Data represent means ± SD (Control, n = 123, *Nkx2.1*^{CreERt2}*; LSL-Kras*^{G12D}, n =167 in 3 fields, respectively). P values (*; P<0.05, **; P<0.01) show the significance with Student's *t*-test, NS, Not Significance

Supplementary Figure 4

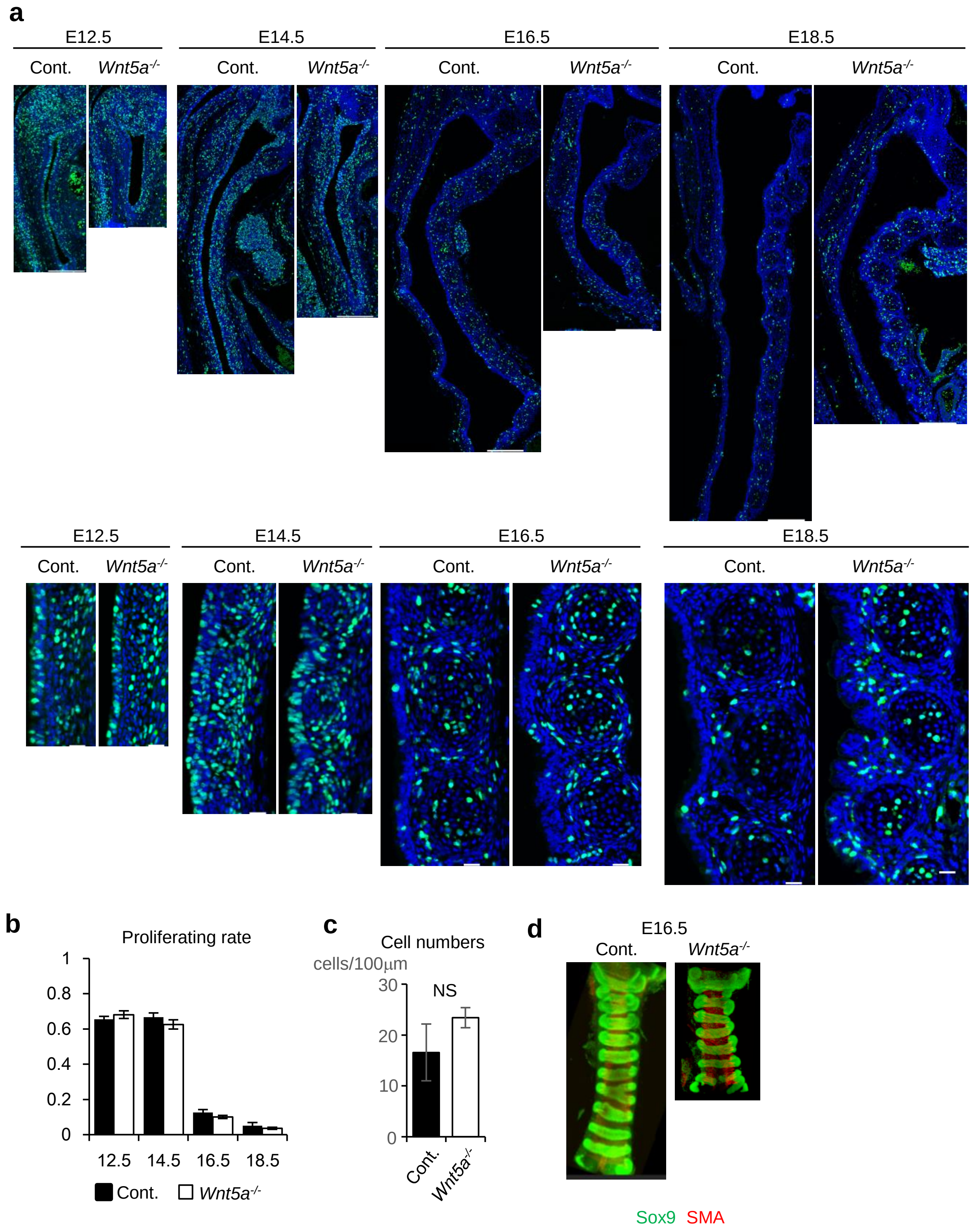

Supplementary Figure 4 Cell proliferation of epithelial cells in developing *Wnt5a*$^{-/-}$ trachea.
(**a**) Distribution of BrdU-incorporated cells in developing trachea of *Wnt5a*$^{-/-}$ mice. Sections were stained for BrdU (*Green*) and TOPRO-3 (*Blue*). Magnified views are shown below. (**b**) Rate of proliferating epithelial cells determined by BrdU-incorporation assay. Each column indicates the mean with SEM (n=3). (**c**) Epithelial cell numbers per 100 µm in *Wnt5a*$^{-/-}$ trachea at E12.5. (**d**) Cartilage morphology in *Wnt5a*$^{-/-}$ trachea and control at E16.5. Whole tracheas were stained for Sox9(*Green*) and SMA (*Red*). D, Dorsal; V, Ventral. Data represent means ± SEM (n≧4). Scale bar; 200 µm (**a**,*upper panel,* **d**), 20 µm (**a;** *Magnified view*).

Supplementary Figure 5

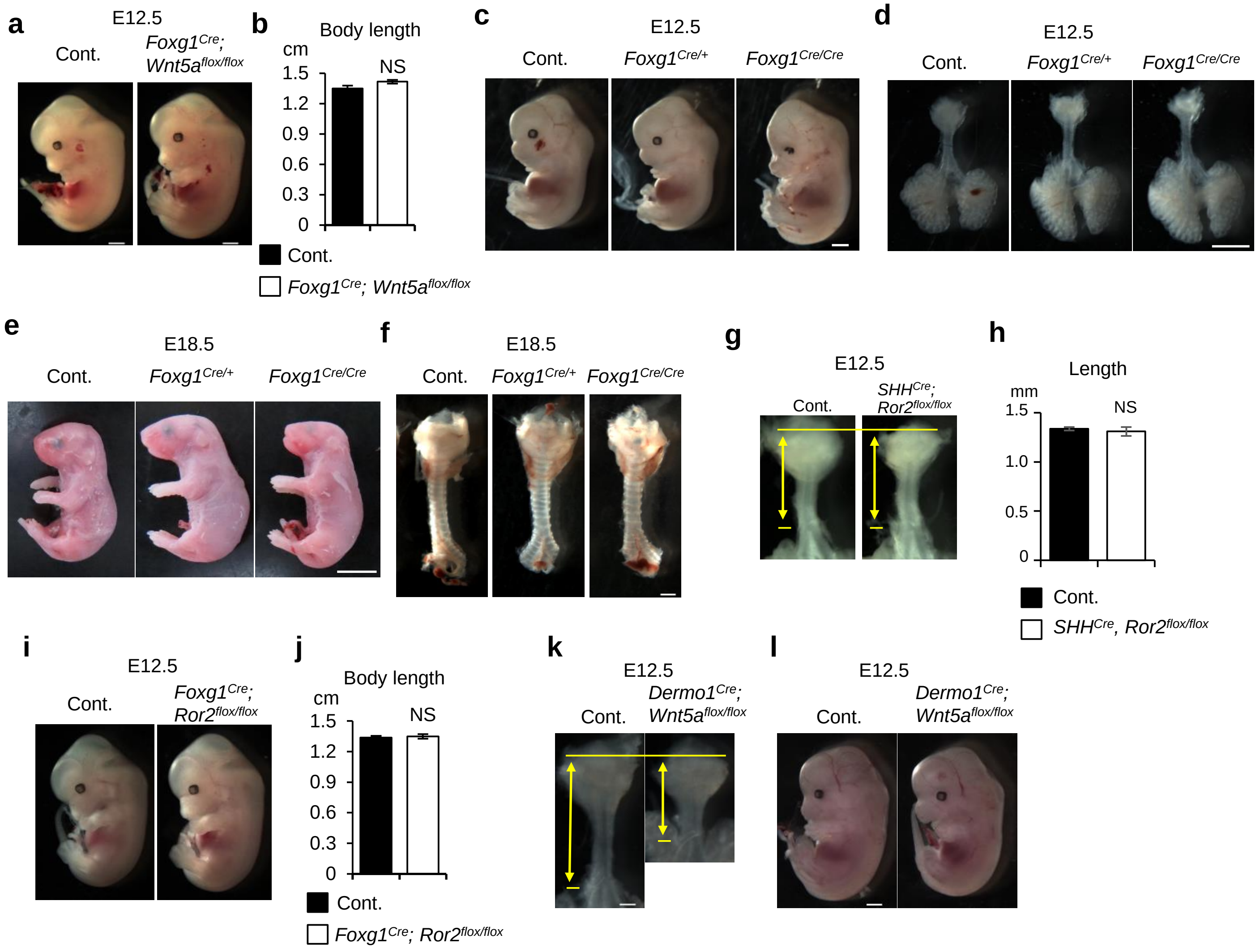

Supplementary Figure 5 Indistinguishable appearance of splanchnic mesodermal Wnt5a or Ror2 mutant embryos from control.
(**a**, **i**, **l**) Gross morphology of *Foxg1*^{Cre}*, Wnt5a*$^{flox/flox}$ (**a**), *Foxg1*^{Cre}*, Ror2*$^{flox/flox}$ (**i**), and *Dermo1*^{Cre}*, Wnt5a*$^{flox/flox}$ (**l**), embryos at E12.5. (**b** and **j**) Body length of *Foxg1*^{Cre}*, Wnt5a*$^{flox/flox}$ (**b**) and *Foxg1*^{Cre}*, Wnt5a*$^{flox/flox}$ (**j**) embryos at E12.5 (Control; n=5, *Foxg1*^{Cre}*; Wnt5a*$^{flox/flox}$ n=6; Control; n=4, *Foxg1*^{Cre}*, Ror2*$^{flox/flox}$; n=3). (**c** and **e**) Gross morphology of *Foxg1*$^{Cre/+}$, and *Foxg1*$^{Cre/Cre}$ embryos at E12.5 (**c**) and 18.5 (**e**). (**d** and **f**) Gross morphology of *Foxg1*$^{Cre/+}$, and *Foxg1*$^{Cre/Cre}$ tracheas at E12.5 (**d**) and 18.5 (**f**). (**g** and **k**) Gross morphology of *SHH*^{Cre}*; Ror2*$^{flox/flox}$ trachea, and *Dermo1*^{Cre}*, Wnt5a*$^{flox/flox}$ (**k**) at E12.5. (**h**) Tracheal tube length (Control; n = 3, *SHH*^{Cre}*, Ror2*$^{flox/flox}$*;* n = 5). NS, Not Significance Scale bar, 1cm (**e**),1mm (**c**, **d**, **l**), 500µm (**f**), 200µm (**k**)

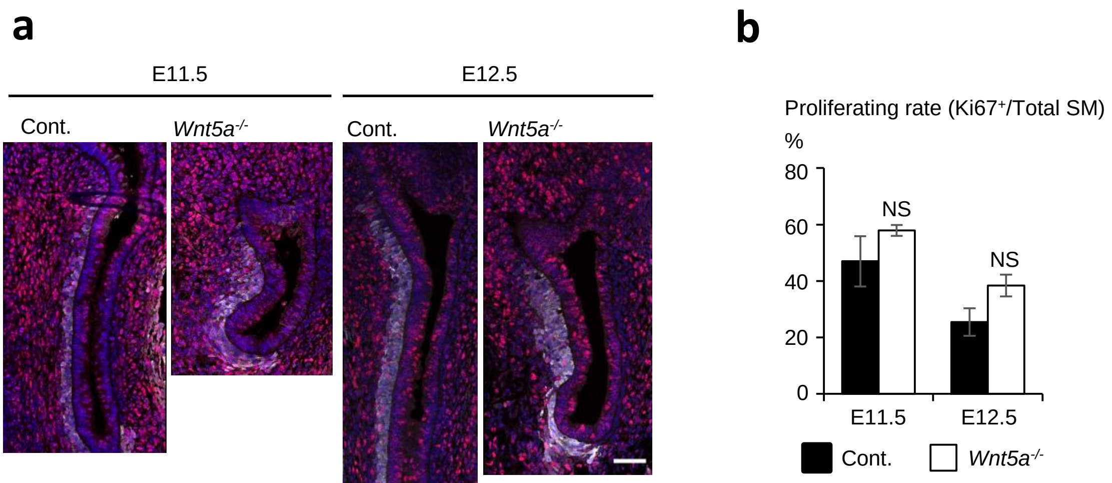

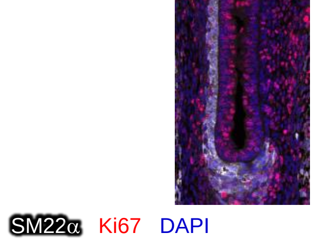

Supplementary Figure 6 Cell proliferation of SM cells in *Wnt5a*$^{-/-}$ trachea during elongation process.
(**a**) Distribution of proliferating Ki67^{+} SM cells in *Wnt5a*$^{-/-}$ trachea and control littermate at E11.5 and 12.5. Sections were stained for Ki67 (*Red*), SM22α (*White*) and DAPI (*Blue*). Magnified views are shown below. (**b**) Proliferating rate of SM cells, which is determined by Ki67 expression. Data represent means ± SEM (n=3). NS; Not Significance. Scale bar; 50 μm

Supplementary Figure 7

a

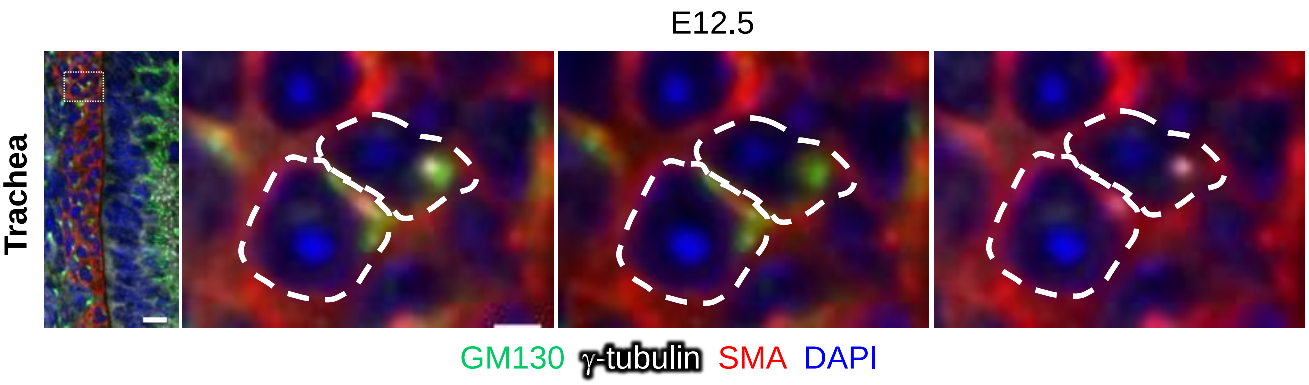

b

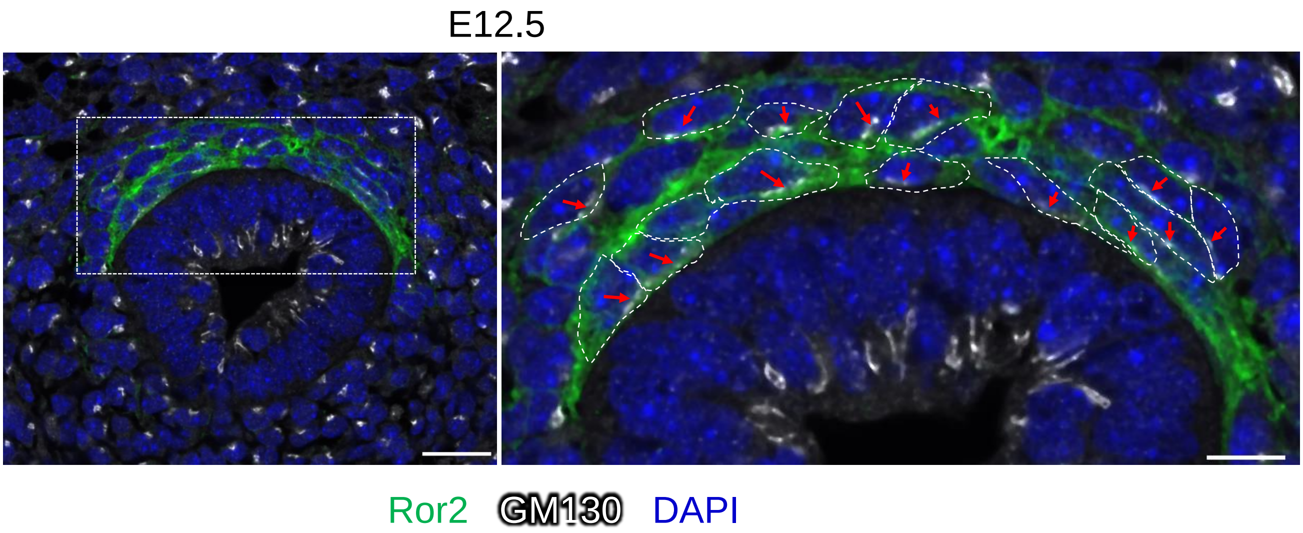

Supplementary Figure 7 Subepithelial cell polarity of smooth muscle cells are determined by the Golgi-apparatus and centrosome positions relative to the nucleus.

(**a**) Smooth muscle cells in subepithelial layer show synchronized cell polarity toward epithelium. Sagittal sections of E12.5 trachea were stained for GM130 (Golgi-apparatus; *Green*), γ-tubulin (Centrosome; *White*), SMA (Smooth muscle; *Red*), and DAPI (nucleus; *Blue*). Scale Bar; 10 μm (Low magnification), 2 μm (High magnification). (**b**) Radial cell polarity toward epithelium. Transversal sections were stained for Ror2 (*Green*), GM130 (*White*), and DAPI (*Blue*). Red arrows indicate the direction of polarity. Scale bar; 20 μm (Low magnification), 10 μm (High magnification).

Supplementary Figure 8

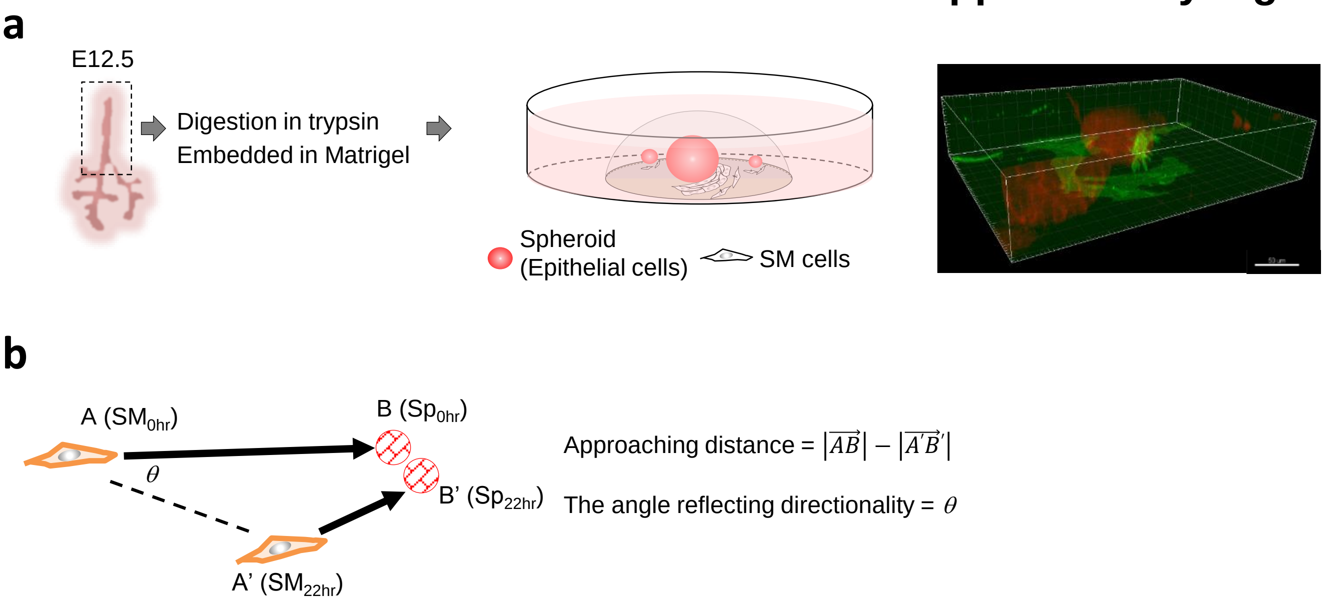

Supplementary Figure 8 Directional migration of SM cells to the epithelium *in vitro*. (**a**) 3D co-culture system of primary SM cells and epithelial cells. (**b**) Quantification of approaching distance and directionality. See Materials and Methods for details.

Supplementary Figure 9

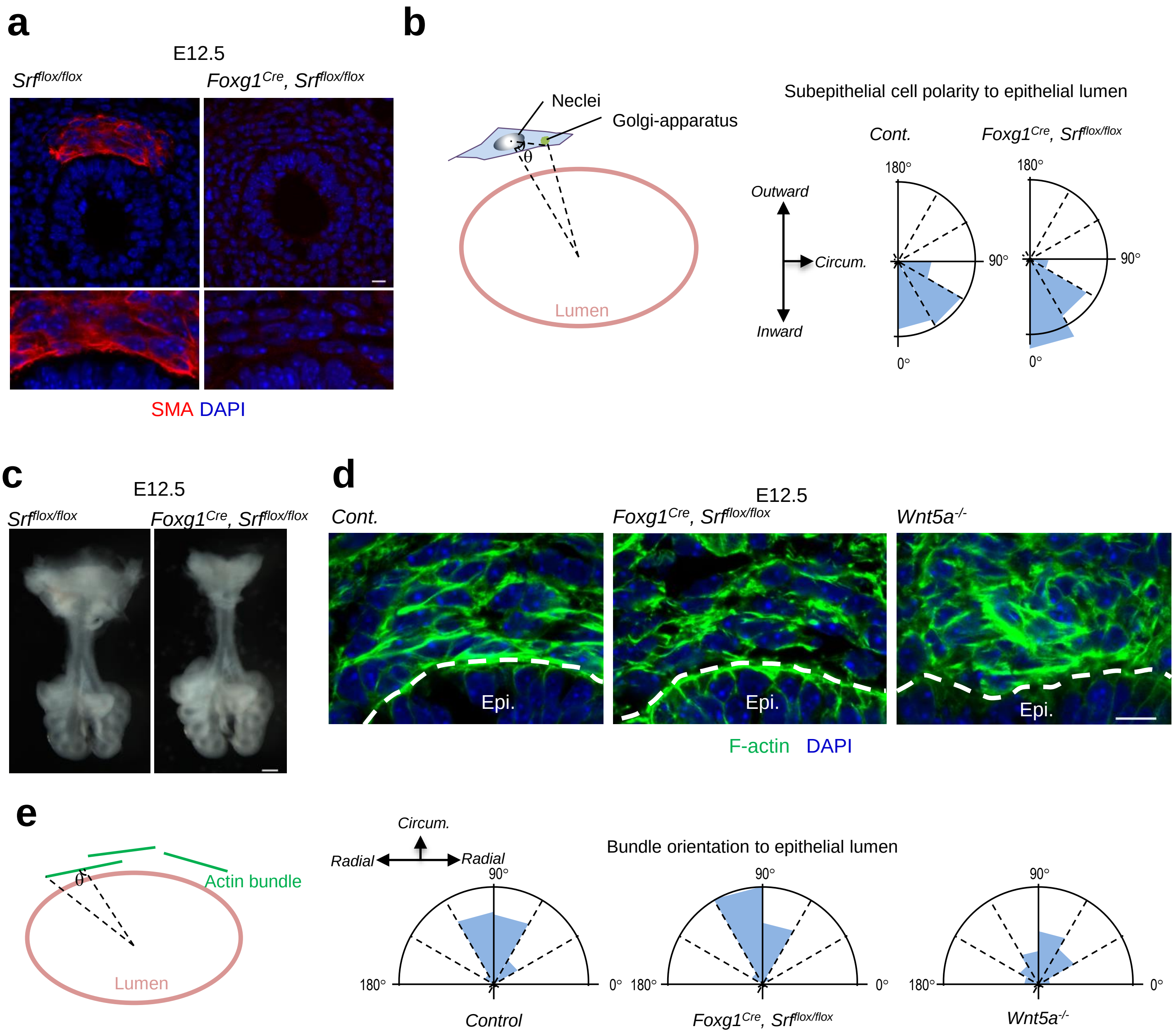

Supplementary Fig. 9 Normal tracheal tube elongation and subepithelial cell polarity in *Foxg1^{Cre}, Srf$^{flox/flox}$* mice.
(**a**) SM morphology in *Foxg1^{Cre}, Srf$^{flox/flox}$* trachea and control at E12.5. Transversal sections were stained for SMA (*Red*), DAPI (*Blue*). Coronal (*Upper*) and transversal (*Lower*) sections. (**b**) QUantification of subepithelial cell polarity, determined by the Golgi-apparatus position relative to the nucleus. (Control; n=26, *Foxg1^{Cre}, Srf$^{flox/flox}$* ; n=29) (**c**) Gross morphology of *Foxg1^{Cre}, Srf$^{flox/flox}$* tracheas and littermate control at E12.5. (**d**) Cell morphology at dorsal subepithelial tissue in *Foxg1^{Cre}, Srf$^{flox/flox}$ and Wnt5a$^{-/-}$ trachea.* Sections were stained for F-actin (*Green*), DAPI (*Blue*). (**e**) Quantification of bundle orientation toward lumen. (Control; n=47, *Foxg1^{Cre}, Srf$^{flox/flox}$* ; n=44, Wnt5a$^{-/-}$; n=50) Epi.; Epithelium, Scale bar, 200 μm (**c**), 10 μm (**a**, **b, d**)

Supplementary Figure 10

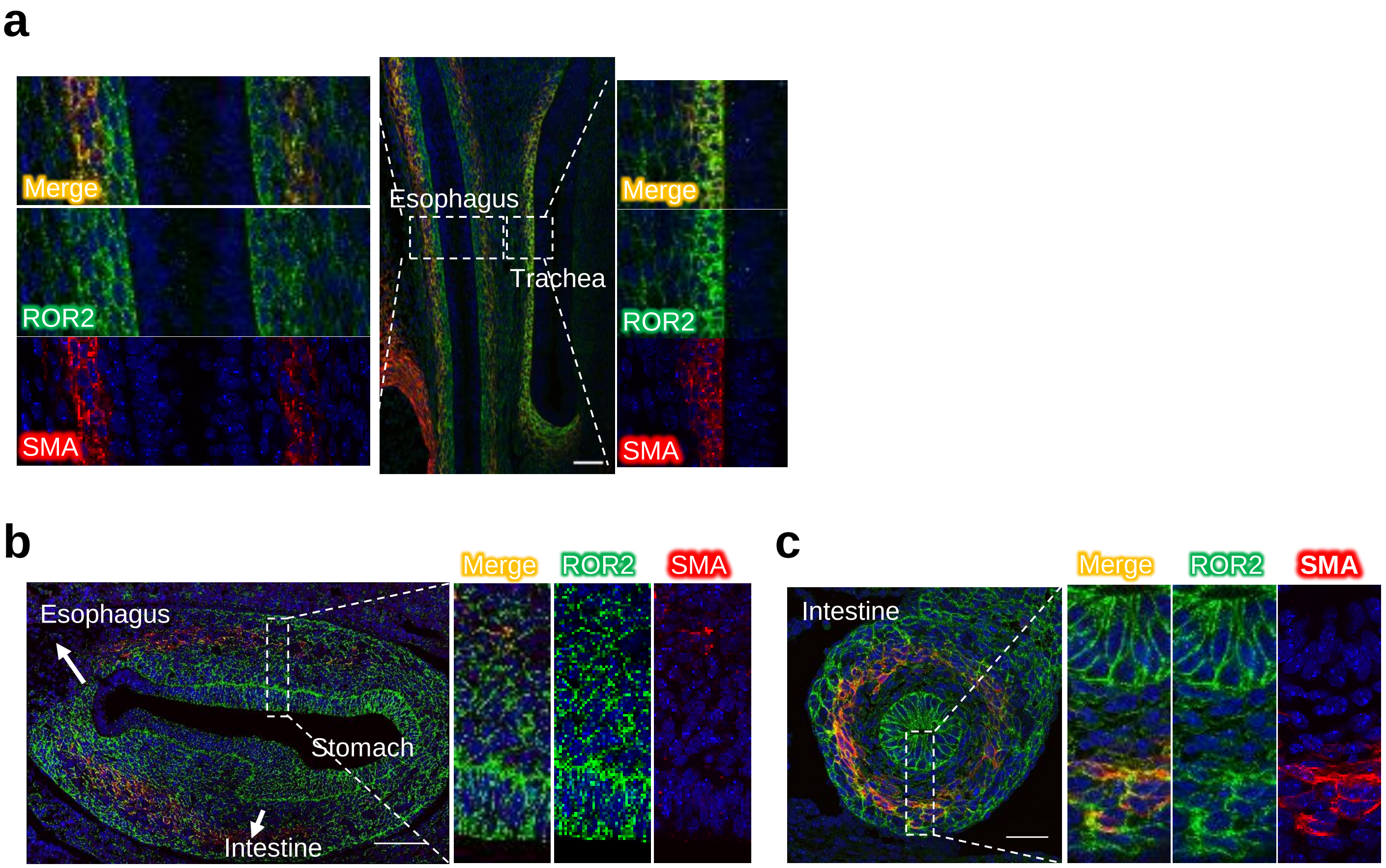

Supplementary Figure 10 The distribution of Ror2 protein in diverse organs derived from foregut endoderm.

(**a-c**) Expression of Ror2 in the esophagus (**a**, **b**) , trachea (**a**), stomach (**b**), and intestine (**c**) at E12.5. Sections were stained for Ror2 (*Green*), SMA (*Red*). Nuclei were counterstained by DAPI (*Blue*). Scale bar; 100μm (**b**), 50μm (**a**), 40μm (**c**).

Supplementary Figure 11

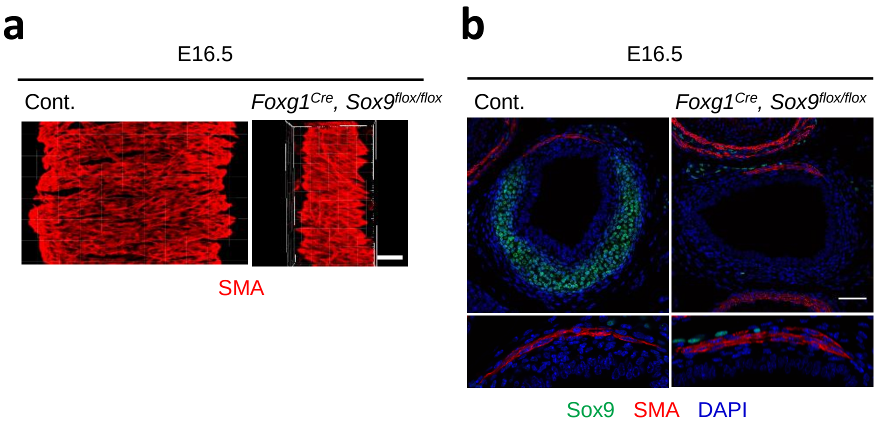

Supplementary Figure 11 Cartilage and SM architecture of the tracheas in *Foxg1^{Cre}, Sox9$^{flox/flox}$* mice. (**a**) SM morphology in *Foxg1^{Cre}, Sox9$^{flox/flox}$* trachea and control at E16.5. Whole tracheas were stained for SMA (*Red*). Coronal sections were shown. (**b**) Cartilage and SM histology of *Foxg1^{Cre}, Sox9$^{flox/flox}$* tracheas. Sections were stained for Sox9 (*Green*), SMA (*Red*) and DAPI (*Blue*). Magnified views are shown below. Scale bar, 50 μm

Supplementary Figure 12

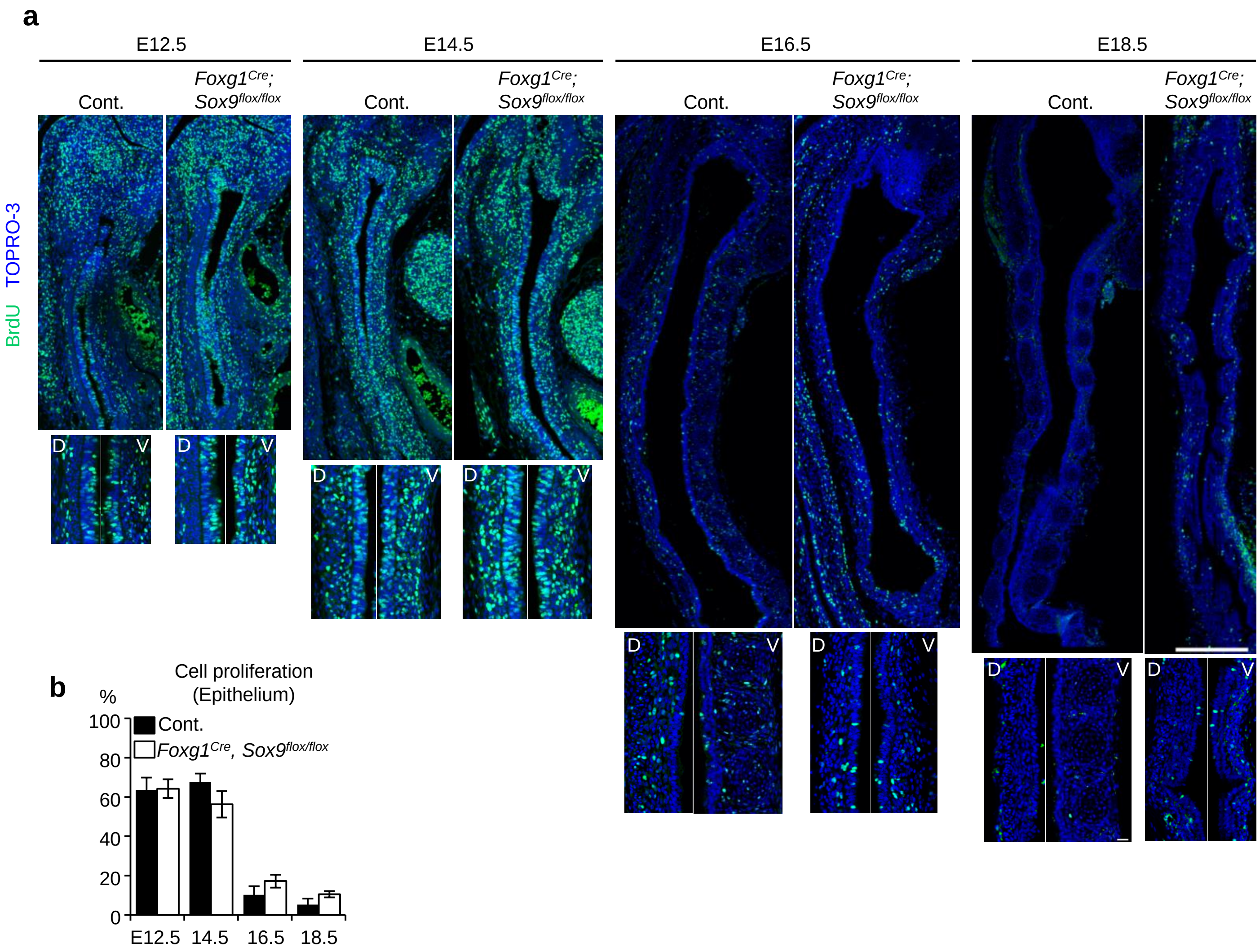

Supplementary Figure 12 Cell proliferation of epithelial cells in *Foxg1^{Cre}; Sox9$^{flox/flox}$* tracheas.
(**a**) Distribution of BrdU-incorporated cells in *Foxg1^{Cre}; Sox9$^{flox/flox}$* tracheas. Sections were stained for BrdU (*Green*) and TOPRO-3 (*Blue*). (**b**) Proportion of proliferating cells determined by cumulative BrdU incorporation assay in *Foxg1^{Cre}; Sox9$^{flox/flox}$* tracheas. (n = 4 or 5). Magnified views are shown below. D, Dorsal; V, Ventral. Data represent means ± SEM (n≧4). Scale bar; 200 μm, 20 μm (*Magnified view*).

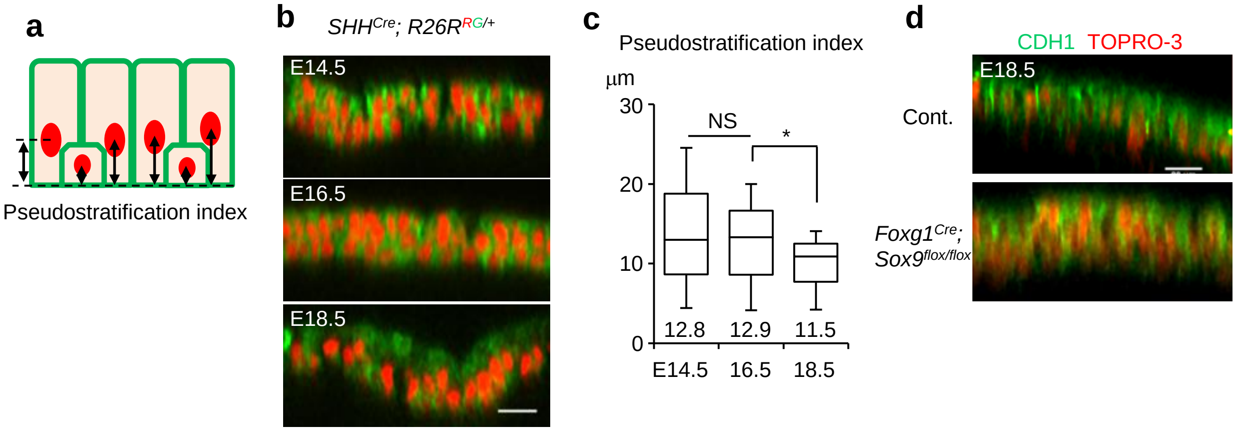

Supplementary Figure 13 Psuedostratification index in *Foxg1*^{Cre}*; Sox9*$^{flox/flox}$ tracheas.
(**a**) Schematic model for calculating the pseudostratification index, the distance between the nuclear centroid to the basement membrane. (**b**) Optical image of the tracheal epithelium in *SHH*^{Cre}*; R26R*$^{RG/+}$ mice*.* (**c**) Pseudostratification index in *SHH*^{Cre}*; R26R*$^{RG/+}$*.* Centre line, box limits and whiskers represent mean, 25 and 75% confidence limits, and min and max values, respectively. Numbers represent means (n = 264, 207, 152 cells in three embryos for E14.5, E16.5, and E18.5). (**d**) Optical image of the tracheal epithelium in *Foxg1*^{Cre}*; Sox9*$^{flox/flox}$. Scale bar: 20 µm.

Supplementary Figure 14

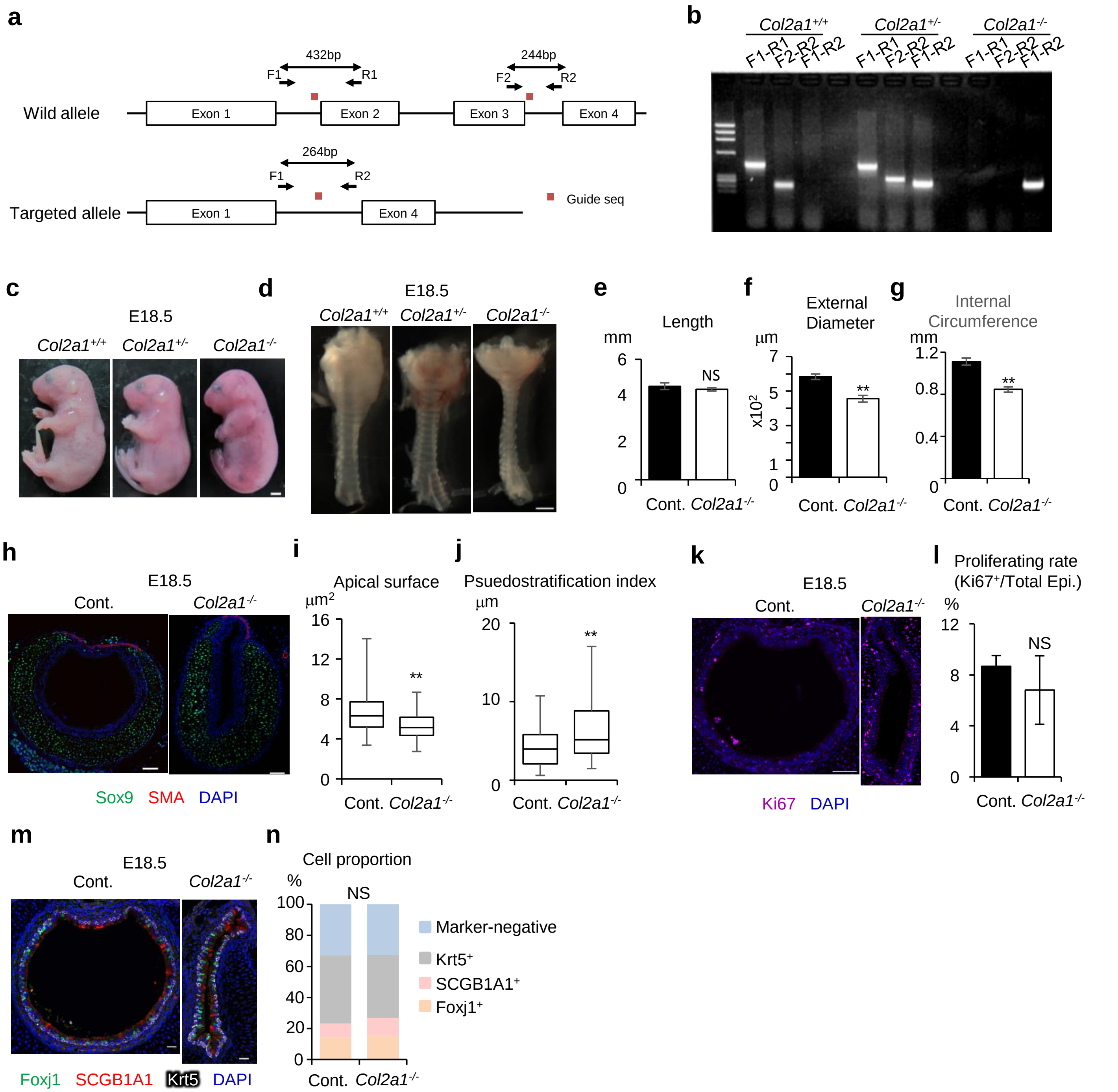

Supplementary Figure 14 Phase 2 trachea tubulogenesis in *Col2a1$^{-/-}$* mice.
(**a**) Generation of *Col2a1* knockout mice with CRISPR/CAS genome editing technology. Guide RNAs were designed at 1st and 3rd intron in order to delete exon2 and exon3. (**b**) Genomic PCR for *Col2a1* genotyping. (**c**) Gross appearance of *Col2a1$^{+/-}$* and *Col2a1$^{-/-}$* mice. (**d - g**) Gross morphology of *Col2a1$^{+/-}$* and *Col2a1$^{-/-}$* tracheas (**d**). Tracheal tube length (**e**), external diameter (**f**) (Control; n = 8, *Col2a1$^{-/-}$* ; n = 9), and internal circumference (**g**) (Control; n = 3, *Col2a1$^{-/-}$* ; n = 3). (**h**) Cartilage and SM architecture of *Col2a1$^{-/-}$* trachea at E18.5. Sections were stained for Sox9 (*Green*), SMA (*Red*) and DAPI (*Blue*). (**i**) Apical surface area of epithelial cells in *Nkx2.1^{mTomato}*, *Col2a1$^{-/-}$* trachea at E18.5. Centre line, box limits and whiskers represent mean, 25 and 75% confidence limits, and min and max values, respectively. (Control; n = 140, *Col2a1$^{-/-}$* ; n = 60 from 3 embryos, respectively) (**j**) Psuedostratification index in *Col2a1$^{-/-}$*. Centre line, box limits and whiskers represent mean, 25 and 75% confidence limits, and min and max values, respectively. (Control; n = 58, *Col2a1$^{-/-}$* ; n = 118 from 2 embryos, respectively). (**k** and **l**) Cell proliferation of epithelial cells in *Col2a1$^{-/-}$* trachea at E18.5. Sections were stained for Ki67 (*Magenda*) and DAPI (*Blue*) (**k**). Proliferating rate of epithelial cells in *Col2a1$^{-/-}$* trachea at E18.5 (**l**) (Control; n = 878, *Col2a1$^{-/-}$* ; n = 672 from 3 embryos, respectively). (**m** and **n**) Cell differentiation of epithelial cells in *Col2a1$^{-/-}$* trachea at E18.5. Sections were stained for Foxj1 (*Green*), SCGB1A1 (*Red*), Krt5 (*Grey*), and DAPI (*Blue*) (**m**). Cell proportion of epithelial cells in *Col2a1$^{-/-}$* trachea at E18.5 (**n**) (Control; n = 826, *Col2a1$^{-/-}$* ; n = 632 from 3 embryos, respectively). P values (*; P<0.05, **; P<0.01) show the significance with Student's *t*-test, NS, Not Significance, Scale bar, 2000 µm (**c**), 500 µm (**d**), 50 µm (**h** and **k**), 20 µm (**m**)

Supplementary Figure 15

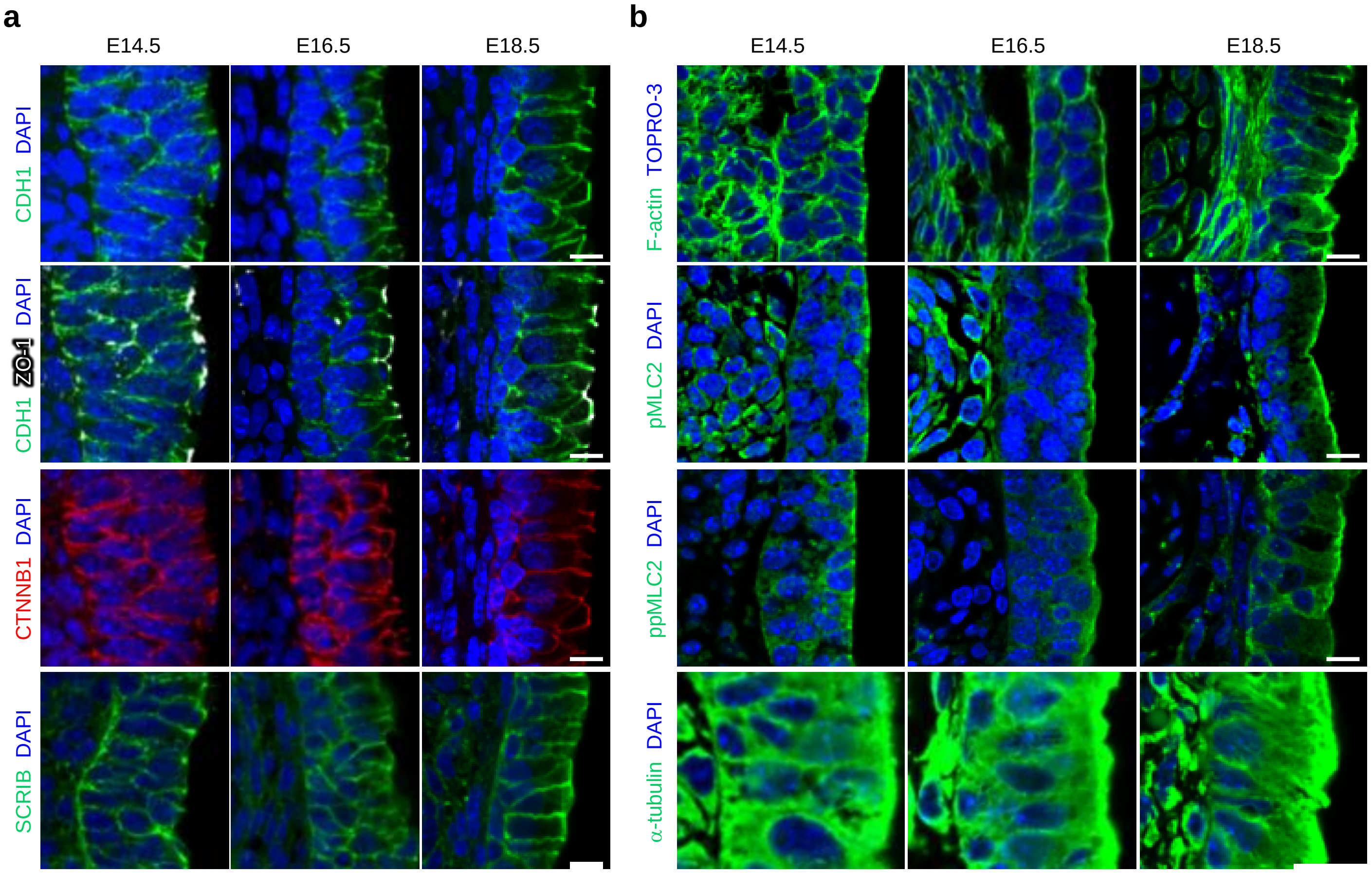

Supplementary Figure 15 Expression and distribution of adhesion-, polarity-, and cytoskeleton-related proteins.
(**a**) Expression and distribution of adhesion- and polarity-related proteins. Sections were stained for CDH1, CTNNB1, ZO-1, or SCRIB. (**b**) Expression and distribution of cytoskeletal components. Sections were stained for F-actin, pMLC^{Ser19}, ppMLC$^{Thr18/Ser19}$, or α-tubulin. Nuclei were counterstained by DAPI or TOPRO-3. Scale bar; 10 μm.

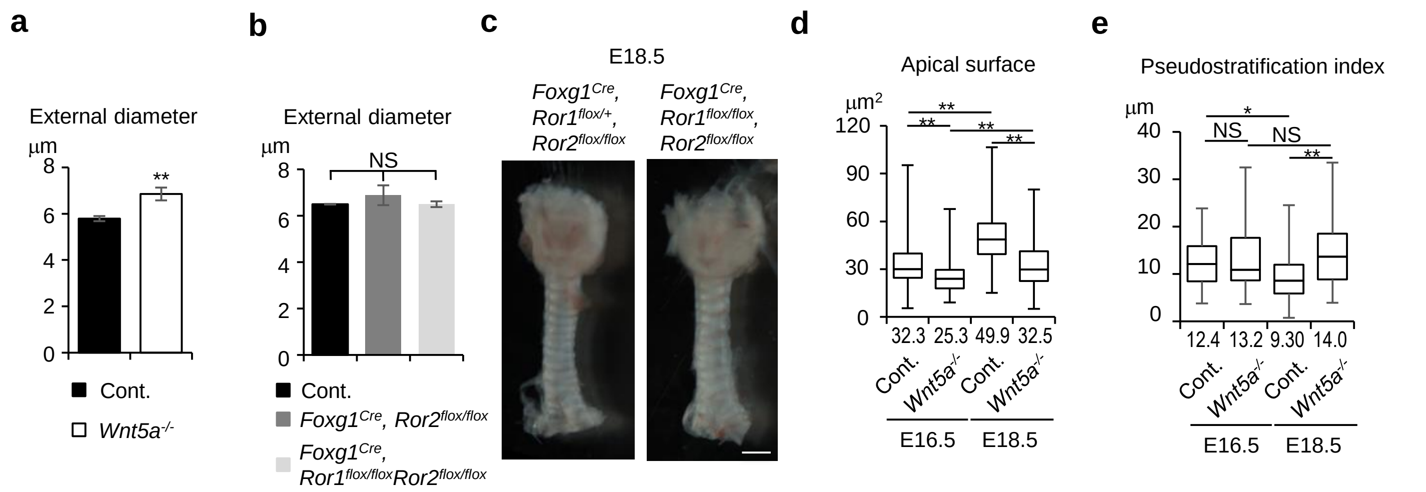

Supplementary Figure 16 Phase 2 tracheal tubulogenesis in Wnt5a/Ror2 mutant mice.
(**a, b**) External diameter of *Wnt5a*$^{-/-}$ (**a**), *Foxg1*^{Cre}, *Ror2*$^{flox/flox}$, *Foxg1*^{Cre}, *Ror1*$^{flox/flox}$, *Ror2*$^{flox/flox}$ trachea (**b**) at E18.5. Data represent means ± SEM (Control; n = 3, *Wnt5a*$^{-/-}$; n = 3, *Foxg1*^{Cre}, *Ror2*$^{flox/flox}$; n = 3, *Foxg1*^{Cre}, *Ror1*$^{flox/flox}$, *Ror2*$^{flox/flox}$; n = 4). (**c**) Gross morphology of *Foxg1*^{Cre}, *Ror1*$^{flox/+}$, *Ror2*$^{flox/flox}$ and *Foxg1*^{Cre}, *Ror1*$^{flox/flox}$, *Ror2*$^{flox/flox}$ trachea at E18.5. (**d**) Apical surface area of epithelial cells in *Wnt5a*$^{-/-}$ trachea at E16.5 and 18.5. Centre line, box limits and whiskers represent mean, 25 and 75% confidence limits, and min and max values, respectively. Numbers represent means (E16.5; Control, n=171, *Wnt5a*$^{-/-}$, n=163, E18.5; Control, n=262, *Wnt5a*$^{-/-}$, n=152). (**e**) Pseudostratification index in *Wnt5a*$^{-/-}$ tracheas at E16.5 and 18.5. Centre line, box limits and whiskers represent mean, 25 and 75% confidence limits, and min and max values, respectively. Numbers represent means (E16.5; Control, n=125, *Wnt5a*$^{-/-}$, n=96, E18.5; Control, n=109, *Wnt5a*$^{-/-}$, n=130). P values (*; P<0.05, **; P<0.01) show the significance with Student's *t*-test (**a**) and Tukey-Kramer method (**b**, **d**, **e**), NS, Not Significance. Scale bar; 500μm
