# Supplementary material for: Synchronized mesenchymal cell polarization and differentiation shape the formation of the murine trachea and esophagus
Source: Nat Commun. 2018 Jul 19;9:2816. doi: 10.1038/s41467-018-05189-2 (PMC6053463; doi:10.1038/s41467-018-05189-2)
Supplement: Supplementary file 3 — Description of Additional Supplementary Files [file 41467_2018_5189_MOESM3_ESM.pdf]

**Description of Additional Supplementary Files**

**File Name**: Supplementary Movie 1
**Description**: 3D reconstruction of luminal structure in whole tracheas. The luminal surface was enlarged throughout embryogenesis, as shown in Fig. 1f.

**File Name:** Supplementary Movie 2
**Description**: 3D reconstruction of epithelial cell clusters in developing trachea. Densely populated epithelial cells rearranged to mature pseudostratified columnar structure by apical enlargement and apical emergence from E14.5 to E18.5, as shown in Fig. 2b.

**File Name:** Supplementary Movie 3
**Description**: 3D reconstruction of SM cell cluster in Wnt5a-/- and control littermate. Individual SM cell extended and aligned in parallel to circumferential axis at E12.5 of normal trachea. In Wnt5a-/-, the cell alignment becomes random and thicker, as shown in Fig. 4b.

**File Name**: Supplementary Movie 4
**Description**: Live imaging of directional SM cell migration in a 3D co-culture system. Live images of migrating SM cells (green) toward epithelial spheres were obtained by two-photon microscopy, as shown in Fig. 4h

**File Name**: Supplementary Movie 5
**Description**: Peristalsis of the trachea in Wnt5a-/- and control littermate. The trachea repeated peristalsis under the ex vivo culture condition, but Wnt5a-/- failed, as shown in Fig. 5a.

**File Name**: Supplementary Movie 6
**Description**: 3D reconstruction of the esophageal SM cell cluster in Wnt5a-/- and control littermate. Individual SM cells extended and aligned in a spiral arrangement at E12.5 in the normal trachea. In Wnt5a-/-, the cell alignment was random.

**File Name**: Supplementary Movie 7
**Description**: 3D reconstruction of epithelial cell cluster in Foxg1Cre; Sox9floxed/floxed and control littermate. Epithelial rearrangement, apical enlargement and apical emergence, was inhibited in Foxg1Cre; Sox9floxed/flox
